# The Impacts of a Clown Doctor Program on an Adolescent Psychiatric Unit: A Mixed Methods Investigation

**DOI:** 10.1007/s10578-023-01545-6

**Published:** 2023-05-25

**Authors:** Glenn Melvin, Jovell Ling Hofmann, Christine Pavlou, Sinh Lu, Stephanie Verstandig, Ashleigh Taylor, Deandra Desilva, Lauren Cameron, Subhadra Evans

**Affiliations:** 1 Centre for Social and Early Emotional Development, School of Psychology, Deakin University, 221 Burwood Highway, Burwood, VIC 3125 Australia; 2 Early in Life Mental Health Service, Monash Health, 246 Clayton Road, Clayton, VIC 3168 Australia; 3 Department of Psychiatry, School of Clinical Sciences at Monash Health, Monash University, Clayton, VIC 3168 Australia

**Keywords:** Clown doctor intervention, Adolescent, Mental illness, Inpatient treatment, Mixed methods

## Abstract

Inpatient psychiatric care may be required to manage adolescents with severe mental health problems. As the ward can be a challenging environment, this study explored the influence of clown doctors on adolescents. Seventy-seven adolescents (13–18 years) and 22 staff from the Monash Health Stepping Stones Adolescent Unit, and 11 clown doctors from The Humour Foundation participated in the study. Bespoke surveys were developed by the research team to collect quantitative self-report data and qualitative responses. Descriptive statistics and thematic analysis suggested that adolescents experienced high levels of fun as well as positive mood during a clown doctor session. Clown doctor programs show promise within an inpatient unit with opportunities for further development being identified. With considerations of the findings, future clown doctor training could include tailoring sessions to the developmental needs of adolescents and developing strategies on how to interact with adolescents who have a mental health disorder.

## Introduction

Adolescents with severe and/or acute mental illness may exhibit heightened risk of suicide [1, 2], and experience acute psychosis or other problems that cannot be effectively managed in outpatient settings, thus warranting a period of psychiatric inpatient care [3, 4]. Nonetheless, whilst the provision of adolescent inpatient treatment is generally effective in minimising the risk or severity of psychopathology, and/or protecting adolescents at a time of crisis or suicidality [4, 5], the environmental setting of inpatient services can be challenging for many, with a considerable number of adolescents and their advocates having voiced negative perceptions and/or experiences regarding their stay at psychiatric inpatient wards [6–12]. While some concerns are difficult to address without changes in policy or appropriate funding (e.g., feelings of restrictiveness caused by physical space), other issues such as boredom [11, 12], and lack of age appropriate services [12] are more feasibly addressed and may improve clinical outcomes. One approach to improving the general experience and wellbeing of children and adolescents during an inpatient stay in general hospital wards is the use of visits by clown doctors. Clown doctors are trained professional performers who work with elements of humour to bring laughter, play and fun to patients as well as their surroundings [13, 14]. The primary goal of clown doctors is to induce a positive change in the immediate environment and emotional state of patients [14, 15]. To achieve this, clown doctors can occupy diverse roles, including medical procedural assistance, distraction and anxiety reduction; providing comfort and emotional support; creating an atmosphere of positivity and warmth; increasing alliance and communication between the patient, caregivers and medical staff [15–17].

In paediatric research, the positive impact of clown doctor interventions has been widely demonstrated in different groups of children across various healthcare settings [13, 16, 18–22]. Positive changes in mood have been reported in a pilot study of eight children with disabilities [20], and positive physiological health-inducing effects in children (*n* = 43) suffering from respiratory pathologies [18]. A systematic review investigating clown doctors as a treatment for preoperative anxiety in children found children in the clown intervention group to report less worries and anxiety symptoms than patients allocated to the control group, with meta-analyses demonstrating an overall positive effect on anxiety [23]. These positive outcomes have been further supported by recent systematic reviews and meta-analyses evaluating controlled trials of clown doctor interventions in medical settings [24, 25], finding significant decreases in anxiety, more pain relief, shortened duration of crying and hospitalisation after operation, and improved psychological wellbeing when compared to controls. Collectively, the findings of these studies emphasise how clown doctors can positively contribute to the physiological, emotional, and behavioural wellbeing of sick children.

Although studies have predominantly focused on the effects of clown doctors on children, clown doctor interventions and their benefits have also been applied to the care of parents with children admitted to a hospital ward [26] or undergoing anaesthesia [27], adult patients with chronic obstructive lung disease [28], pregnant women [29], as well as elders with dementia [30–32]. Notably, a study of adult patients hospitalised in a psychiatric ward showed a significant decrease in disruptive behaviors (e.g., attempted escapes, fighting) and self-injury during a clown intervention period compared to baseline, suggesting a positive impact of ward clown activities [33]. The benefits of clown doctors have also been extended to clinical and nursing staff, including nurses in a children’s rehabilitation hospital [34] and doctors, nurses, and technicians in an oncology ward [35]. Nurses reported a lowered negative mood and a positive difference on their role in playing and communicating with patients [33] as well as positive changes to the atmosphere in the ward, relieving stress and improving mood for medical staff [35].

Despite the encouraging findings of the benefits of clown doctor programs for both patients (children, adults, and elderly) and staff across a range of settings, to the authors’ knowledge, no prior study has investigated the impact of a clown doctor program in an adolescent inpatient psychiatric setting. Inpatient psychiatric units are distinct from general medical units in that patients have been admitted due to a range of psychiatric disorders, many of which impair their capacity to interact with others, and extremes in emotion (e.g., sadness, despair, euphoria) and behaviour (e.g., aggressive, manic, disorganised) may be evident, both of which may challenge the task of clown doctors. More broadly, as adolescence is also a formative period of growth with many developmental changes that distinctively separate them from any other age group, it is important to understand whether the provision of clown doctor is age-appropriate and beneficial for inpatients of this cohort.

This study was therefore the first to investigate whether clown doctor visits have a positive impact on the mood and experiences of adolescents residing in a psychiatric inpatient unit within a hospital. We were interested in evaluating adolescents’ reported levels of fun and interaction with the clown doctors, as well as their general feedback about what they liked, disliked and/or needed improvement. To gain further insight into clowning as an adolescent inpatient intervention, we were also interested in the clown doctors’ and clinical staffs’ perspectives about the adolescents during the intervention and the overall visit. Overall, the aim was to provide an overview of how the clown doctor visits were experienced and perceived by adolescents, clown doctors and clinical staff in order to provide practical guidelines for the further development of clown doctor programs for this population.

## Method

### Design

This mixed-method study evaluated the perceived experiences of adolescent inpatients, clown doctors and clinical staff regarding clown doctor visits as an intervention program. This study was uncontrolled and study variables were assessed at single time points. Qualitative data from open-ended questions were triangulated with quantitative survey data to provide a richer account of participant and observer experiences.

### Research Setting

The research was conducted at the Stepping Stones Adolescent Inpatient Psychiatric Unit, Monash Children’s Hospital in Victoria Australia. Stepping Stones is a 15 bed inpatient and five transition (day) bed Unit that provides mental health assessment and treatment interventions to adolescents (aged 12 to 17 years) who present with acute and severe mental health problems, associated high risk factors and have difficulty maintaining their own safety and the safety of others. During data collection, the average length of stay for inpatients at Stepping Stones Adolescent Inpatient Psychiatric Unit was 7.4 days (*SD* = 0.7).

### Participants and Sampling

Participants were recruited via convenience sampling and comprised adolescents hospitalised in the psychiatric ward of Stepping Stones. To be eligible for the study, adolescents had to be admitted for the treatment of a mental health illness or disorder but were not viewed by clinical staff as being unfit to participate due to their current mental state (e.g., agitated, aggressive or visibly distressed at the time of the clown doctor visit). Adolescents were excluded from the study if they had a condition that might impair their capacity to voluntarily participate (e.g., active psychosis, intellectual disability, severe intoxication or acute suicidal risk) and/or did not speak English. All unit staff and clown doctors were considered eligible to participate. Nurses, teachers, medical personnel, and allied health staff from speech pathology, social work, and psychology were recruited from Stepping Stones and clown doctors were recruited from The Humour Foundation, a charity organisation dedicated to promoting and delivering the health benefits of humour.

### Intervention

Every two weeks, clown doctors from the Humour Foundation visited inpatients at the Stepping Stones adolescent inpatient psychiatric unit. During each session of the Clown Doctor program, the clown doctor(s) attempted to engage with adolescents in various ways to promote play, laughter and fun, including the use of humour therapy to lighten the hospital atmosphere as well as telling jokes, singing songs, playing music and performing magic tricks. Sessions were 45 min in duration.

### Instruments

The research team reviewed the literature prior to the study. Due to the absence of appropriate measures, the research team together with clown doctors, developed bespoke measures focusing on the experiences of adolescents, clown doctors, and clinical staff during a clown doctor session in a psychiatric context. Three surveys were developed for adolescents, clown doctors and clinical staff.

**The Clown Doctor Inventory- Young Person Report.** The survey comprised of 16 self-report items, beginning with two demographic questions asking about age and gender and one question identifying how many times the adolescents had been visited by the clown doctor(s), rated on a four-point scale from ‘1’ to ‘more than 5’. The survey followed with six questions examining the adolescents’ perceived levels of fun, interaction and distraction during the clown doctor session(s), rated on a four-point scale from ‘no’ to ‘a lot’. Two further questions explored the adolescents’ mood and the feeling of the room following the clown doctor visit, rated on a five-point scale with responses ranging from ‘much worse’ to ‘much better’. The last four open-ended questions explored what made the clown doctor visit fun, what the adolescents liked and/or disliked, and what improvements could be made to future clown doctor sessions. The internal consistency of the inventory was found to be high (α = 0.84).

**The Clown Doctor Inventory- Clown Doctor Report.** The survey comprised of seven self-report items. Four questions investigated the clown doctors’ perception of the participants’ experience of fun, interaction, and the general success of a single clown doctor session. The ratings were on a four-point scale ranging from ‘no’ to ‘a lot’. One question investigated whether there were any perceived changes in the feeling of the room, rated on a five-point scale with responses ranging from ‘much worse’ to ‘much better’. The last two open-ended questions asked clown doctors to list techniques, if any, that successfully engaged and did not successfully engage adolescents. The internal consistency of the inventory was found to be acceptable (α = 0.73).

**The Clown Doctor Inventory – Unit Staff Report.** The survey comprised of eight self-report items. Unit staff were asked about the frequency of clown doctor visits they had participated in over the last six months, with ordinal responses of ‘1 to 5’, ‘6 to 10’ and ‘11+’. Four questions investigated the staffs’ perception of the adolescents’ level of fun and interaction with the clown doctors, how beneficial the clown doctor session(s) were for the adolescents, and whether the clown doctor visits assisted them to observe new behaviours in the adolescents. The ratings were on a four-point scale ranging from ‘no’ to ‘a lot’. Staff were asked about the room’s milieu, whether change occurred during the clown doctor visit, on a five-point scale ranging from ‘much worse’ to ‘much better’. Staff also responded, with a binary response of either ‘yes’ or ‘no’, to whether they would recommend other units like Stepping Stones who don’t have clown doctors to start using them, and whether they thought clown doctor visits assisted them in any way in assessing a young person. The internal consistency of the inventory was found to be acceptable (α = .71).

### Procedure

Data collection began in July 2018 and ceased in September 2019. Following parental or guardian consent and the experience of a clown doctor session, members of the research team approached eligible adolescent participants and gave a verbal explanation of the project, an opportunity to ask questions as well as an explanatory sheet, consent form and questionnaire. On a voluntary and anonymous basis, adolescents completed the Clown Doctor Inventory- Young Person Report. Clown doctors and clinical staff were informed about the project by the investigators during staff meetings and also via a flier on staff noticeboards. Clown doctors and clinical staff received an information sheet, consent form and relevant questionnaire. All questionnaires were short in duration, administered via paper-and-pencil format, and were completed immediately following the clown doctor session.

Some participants (patients and clown doctors) completed the inventory on multiple occasions, however only the responses to their first survey were used for analysis in this study.

### Data Analysis

Descriptive statistics, including measures of central tendency and frequency, were used to report quantitative findings of the survey. For the qualitative open-ended survey data, a critical realist perspective was used, which assumes there is a reality to observe and describe but is not independent of the researcher’s viewpoint [36, 37]. Qualitative data were examined according to Braun and Clarke’s [38, 39] six-step reflexive thematic analysis. Open-ended questions were designed to understand adolescents experience using preconceived notions considered to be relevant to interaction with the clown doctors, including experiences of fun, likes, dislikes and opportunities for improvement suggesting a deductive approach. However, given the lack of prior studies examining adolescent’s experience of a clown doctor program, our approach was also informed by an inductive approach, which allows the data to speak for itself with themes closely connected to the data. Thus, the approach to analysis was both inductive and deductive. Analysis involved identifying themes within and between the open-ended answers, with codes developed from the data rather than an existing framework. The six stages of thematic analysis used in the study were: (i) data familiarisation; (ii) code creation; (iii) theme search; (iv) theme review; (v) theme description and naming; and (vi) report preparation. With regards to reflexivity, which is an awareness and self-appraisal of the researcher’s experience and position within the research project [40], the researchers had expertise in clinical psychology (GM), developmental psychology (SE, GM, JLH), mental health nursing and inpatient clinical care (CP, SV, AT, DD) and all but one (SE) had observed Clown Doctor sessions. Thus, the research team’s experience provided a range of first-hand and multi-disciplinary perspectives relevant to clown doctoring. Some researchers were mental health nurses involved in the care of the participants, study conceptualisation and data collection but did not participate in data analysis (CP, SV, AT, DD). To reduce bias, researchers involved in qualitative data analysis (JLH, GM, SE) held data review meetings and consensus was reached as to the relevant themes and subthemes.

## Results

The final sample included 77 adolescents aged between 13 and 18 years (*M* = 16.05 years, *SD* = 1.29 years), with majority of adolescent participants being female (66%). A total of 11 clown doctors from The Humour Foundation delivered the Clown Doctor program to patients at Stepping Stones, and 22 clinical staff from Stepping Stones participated in the present study.

The descriptive statistics for adolescent responses to the surveys are reported in Table 1.

**Table 1:** Adolescent Responses to the Young Person Clown Doctor Inventory

Questions	N	No *n* (%)		A little *n* (%)		Somewhat *n* (%)		A lot *n* (%)	
Did you have fun during the clown doctor visit?	77	4 (5.2%)		16 (20.8%)		36 (46.8%)		21 (27.3%)	
Is a day with a clown doctor visit more fun than a day without a clown doctor visit?	73	11 (15.1%)		17 (23.3%)		26 (35.6%)		19 (26.0%)	
Did you interact with the clown doctors?	77	2 (2.6%)		21 (27.3%)		21 (27.3%)		33 (42.9%)	
Did you interact with other young people during the clown doctor visit?	77	4 (5.2%)		24 (31.2%)		28 (36.4%)		21 (27.3%)	
Did you enjoy your interactions with the clown doctors?	77	3 (3.9%)		19 (24.7%)		30 (39.0%)		25 (32.5%)	
Did the clown doctor visit distract you from any problems you might be having?	77	14 (18.4%)		22 (28.9%)		20 (26.3%)		20 (26.3%)	
	N	Much Worse	Worse		The same		Better		Much Better
		*n* (%)	*n* (%)		*n* (%)		*n* (%)		*n* (%)
How did the clown doctor visit affect your mood?	77	-	-		37 (48.1%)		29 (37.7%)		11 (14.3%)
How did the clown doctor visit change the feeling in the room?	73	-	1 (1.3%)		20 (26.3%)		43 (56.6%)		12 (15.8%)

*Note. N* = sample size, *n* = frequency

Data presented consist of responses to surveys following the adolescent’s first clown doctor session. At the time of participation, 44% (n = 34) of adolescents reported having experienced more than one visit from the clown doctors during their admission. As illustrated in Table 1, approximately 95% (n = 73) of adolescents reported they had fun during the clown doctor session, with 27% (n = 21) stating they experienced ‘a lot’ of fun. Furthermore, 85% (n = 62) of adolescents responded they have more fun on a day with clown doctors present than absent. Only 2.6% (n = 2) of adolescents reported having no interaction with the clown doctors, and approximately 96% (n = 74) of the adolescent sample indicated that they enjoyed their interactions with the clown doctors. Approximately 52% (n = 40) of adolescents reported being ‘somewhat’ or more distracted from problems they might be experiencing.

As illustrated in Table 1, no adolescents reported that the clown doctor had a negative impact on their mood – instead, 52% (n = 40) of adolescents reported ‘better’ and ‘much better’ moods after the clown doctor session(s). Furthermore, the feeling of the room after clown doctor session was reported ‘better’ to ‘much better’ by 72% (n = 55) of adolescents, with only one participant reporting the feeling to be ‘worse’.

Descriptive statistics of clinical staff responses to the survey are presented in Table 2.

**Table 2:** Clinical Staff Responses to Measures of Adolescent Fun, Interaction with Clown Doctors, Observation of New Behaviours and Benefits of Visits

Questions	N	No *n* (%)	A little *n* (%)	Somewhat *n* (%)	A lot *n* (%)
In general, do you think the young people have fun during the clown doctor visits?	22	-	4 (18.2%)	9 (40.9%)	9 (40.9%)
In general, do the young people interact with the clown doctors during the visits?	21	-	2 (9.1%)	13 (61.9%)	6 (28.6%)
Do the clown doctor visits assist you to observe new behaviours in the young people?	21	5 (23.8%)	2 (9.5%)	11 (52.4%)	3 (14.3%)
Do you believe that the clown doctor visits are beneficial for the young people?	21	1 (4.8%)	4 (19.0%)	7 (33.3%)	9 (42.9%)

*Note. N* = sample size, *n* = frequency

As illustrated in Table 2, all clinical staff members reported young people had fun and interacted with clown doctors during their visits. Approximately 66% of clinical staff reported clown doctors ‘somewhat’ to ‘a lot’ assisted them in assessing new behaviours in young people. Further, 96% of clinical staff believe clown doctors were beneficial to young people.

Overall, clinical staff reported positive changes in milieu, with 76% of staff reporting the room being ‘better’ (n = 14; 57%) or ‘much better’ (n = 4; 19%) following a visit. Some staff reported the room being ‘the same’ (n = 2; 9.5%) and some reported the room being ‘worse’ (n = 2; 9.5%) or ‘much worse’ (n = 1; 5%) following the clown doctor visits.

Finally, approximately half of the staff reported that the clown doctor visits assisted them in assessing a young person (n = 11; 52%) and nearly all clinical staff reported that they would recommend similar inpatient psychiatric units use clown doctors if they don’t already (n = 18; 90%).

The descriptive statistics for clown doctor responses to the surveys are presented in Table 3.

**Table 3:** Clown Doctor Responses to Measures of Adolescent Fun, Willingness to Interact, Openness to Session, Room Feel and Session Success

Questions	No *n* (%)	A little *n* (%)	Somewhat *n* (%)	A lot *n* (%)
Did the young people have fun?	-	-	5 (45.5%)	6 (54.5%)
In general, did the young people interact with the clown doctor’s visit?	-	1 (9.1%)	3 (27.3%)	7 (63.6%)
At the end of the session, were the young people in general more open to social interaction than at the beginning?	-	-	6 (54.5%)	5 (45.5%)
Overall, was it a successful visit?	-	-	3 (27.3%)	8 (72.7%)

*Note. N* = 11

As illustrated in Table 3, all clown doctors reported young people had ‘somewhat’ to ‘a lot’ of fun, interaction and improved openness to social interaction. Every clown doctor highlighted the visit as a success. Every clown doctor reported ‘better’ (n = 9; 82%) to ‘much better’ (n = 2; 18%) feeling in the room following a visit.

### Qualitative Findings

Of the 77 participants, 76 provided qualitative data. Within the survey data, three themes were generated, including how the sessions made adolescents feel (*The experience was ‘fresh air’*), a comment on the content and structural aspects of the sessions that did and did not work *(“Playing with the stretchy banana”: Dissecting the experience of fun*), and the importance of considering the developmental needs of this group (*Developmental appropriateness: “They tried a little too hard”*). Generally, clown doctors’ comments verified adolescents’ feelings and perceptions.

#### The Experience was “Fresh Air”

The first theme related to how the clown doctor experience made adolescents feel. For the most part, adolescents reported benefits in the form of improved mood and a sense of joy through laughter. Adolescents appreciated the way that the sessions meant a welcome distraction from their problems and allowed for an increased connection to others.

In terms of improved mood, clown doctor sessions gave adolescents the opportunity to laugh and improve their mood. As expressed by participant 17, 15-year-old male: “managed to make a lot of us smile and laugh,” while another reported: “something different to do, it makes me a bit happier.” (Participant 53, 17-year-old female). Generally, there was a sense of lightness or improved atmosphere on the wards. Adolescents found they enjoyed seeing other participants having an improved mood during the sessions, and they appreciated the change in atmosphere: “there was lots of laughs which was a good change from the usual quiet in the ward” (Participant 5, 17-year-old female).

Speaking to a possible mechanism for why the clown doctor sessions seemed to improve their mood, adolescents expressed being able to shift their attention away from their problems as a fun aspect of the sessions: “This fun experience distracted us from all of our thinking and made us laugh and have fun” (Participant 41, 16-year-old male).

Adolescents also expressed the benefit of increased human connection through the experience. They conveyed a sense of enjoyment talking to the clown doctors and were able to develop connections and build a relationship with them, “talking about things outside of the ward with a really personable, friendly person” (Participant 16, 18-year-old female). Some highlighted that it was fun being able to talk to someone other than a nurse or doctor on the ward. Clown doctors expressed that building rapport was a key aspect of the sessions. This occurred through personal conversations “being interested in what they had to say” (Clown doctor 11) and chatting about things removed from the ward that were important to adolescents, including “discussion about hobbies and past times, discussion about pets” (Clown doctor 1). Through such communication, clown doctors were actively able to build relationships with adolescents during the sessions.

Not all participants reported psychological benefits from the sessions. For some, there was a sense of sensory overload from the noise of the sessions. This sensory overload may have proven overwhelming and chaotic for some adolescents: “the noise and yelling/noises not just from the clowns though” (Participant 76, 15-year-old male). Clown doctors confirmed that some activities and games could be overstimulating “perhaps getting one girl too stimulated to the point of being a little boisterous” (Clown doctor 1). Clown doctoring may need to be modified to accommodate the sensory needs of young people who can become over-stimulated (e.g., having a fun but quiet corner).

#### “Playing with the Stretchy Banana”: Dissecting the Experience of Fun

There were two aspects of the sessions that adolescents reported that they appreciated or could be improved, including the content and structure of the sessions. Content that was enjoyable included props and activities, which were seen as fun and well-liked. One particularly popular prop was the use of bubbles, with the majority of adolescents highlighting positive interactions with bubbles. Other props such as balloons and miscellaneous objects including a “stretchy banana” (Participant 57, 16-year-old female) were also part of the session and enjoyed by adolescents. ‘Silly’ games and activities were also favoured, including table tennis and playing music. The high spirits and sense of fun that clown doctors brought to a range of activities appeared contagious: “The way the doctors joked around us and changed their humour to ours” (Participant 62, 16-year-old female). Clown doctors confirmed their props were successful in engaging adolescents. They reported using “musical instruments… magic paper bag tricks” (Clown doctor 1), “puppetry” (Clown doctor 9), and “bubbles” (Clown doctor 4 and 7) to good effect. In terms of games and activities, playing table tennis with the back of the ukulele was reported as a successful tool. Clown doctors expressed that performance-based acts such as magic tricks and telling jokes were effective tools to engage with adolescents. Music, including a mass sing-along was emphasised by some clown doctors: “One girl sang two songs beautifully. A guy sang and played two on the guitar. Big group singalong to ‘riptide’. Some shy kids joined in, chef got involved, huge applause.” (Clown doctor 10). In contrast to successful acts, “excessive farting” (Clown doctor 5) or “fart routine” (Clown doctor 7) were noted as being unsuccessful.

Adolescents shared a number of ideas for how to improve the experience of props and activities, including increasing the amount of activities and props brought in by clown doctors. Participant 17, (15-year-old male) wrote “try to be more creative or innovative – show us stuff we haven’t seen”. Several male participants reported a desire to have more activities that included physical activity within clown doctor sessions, for example, “maybe if we did some actual activities like basketball, volleyball, cooling off and board games and table tennis” (Participant 26, 16-year-old male).

The second aspect of the sessions that participants appreciated related to session structure. Adolescents enjoyed how the clown doctors gave them a chance to do things on their own, demonstrating a sense of respect and autonomy granting on the part of the clown doctors. Viewed as a positive aspect of the session structure, adolescents were not forced to participate if they did not want to. Participant 5 (17-year-old female) wrote “they interacted with everyone even the kids in HDU [High Dependency Unit] but if they saw someone didn’t feel like interacting they left them alone”. Overall, adolescents felt a sense of inclusion. They were able to come together and interact as a group, with adolescents they might not have had a chance to interact with on the ward. Participant 56 (17-year-old female) wrote “I like that they include everyone in the stuff they are doing”. Clown doctors expressed the layout deliberately encouraged inclusion, for example “the use of balloons encouraged social interaction and sharing” (Clown doctor 3). Encouraging interaction and sharing was noted as a positive technique used to engage with adolescents.

However, not all adolescents had an experience of inclusion. Participant 52 (16-year-old female) wrote “sitting not so close to be disincluded [sic.] and not sharing around activities”. Some adolescents did not feel like they had a chance to do all the activities, while others reported they were separated from other adolescents when interacting with a clown doctor. Some clown doctors highlighted the organisation of the sessions could contribute to this, where focused interaction with one adolescent can be a negative technique, “don’t use too much on an intense focus, don’t chat to just one person” (Clown doctor 11). One reported that adolescents were overwhelmed with the attention, causing them to withdraw. Clown doctor 8 reported “sometimes focusing on one young person makes them withdraw, better to start as a group.”

Adolescents provided recommendations for improving the structure of future clown doctor sessions, mostly centred around ideas for increased access, including increasing visit frequency, visit length and more clown doctors. Several adolescents expressed that they enjoyed everything about the clown doctor sessions and did not want to change anything.

#### Developmental Appropriateness: “They Tried a Little Too Hard”

Adolescents highlighted both positive and negative aspects of the suitability of the clown doctor session for a group of adolescents. Adolescents expressed they appreciated and found it fun when they were able to speak to the clown doctors without them being immature, “when they stop acting really childlike, you can actually develop a proper conversation” (Participant 60, 16-year-old female). However, adolescents knew when clown doctors were trying too hard to fit in or make them laugh, which left them feeling uncomfortable and awkward. For example, Participant 3 (15-year-old male) wrote “too cliché” when they were forcing interaction. Participant 47 (15-year-old female) wrote “I didn’t like how they tried a little too hard to join in on things we were already doing, not stepping back after”.

Some adolescents felt the clown doctor session content and humour were not appropriate for their age group and they felt like they were being treated as children. One adolescent highlighted they would prefer “less clowny costumes” (Participant 34, 13-year-old female), while another felt that “the only thing that I found annoying was that I felt like they were treating or acting like their audience were a lot younger than they were” (Participant 48, 17-year-old male). Similarly, clown doctor 6 highlighted an ineffective method of engagement, “anything a bit ‘young’ - like squeaks was a bit confusing maybe and not so effective.” Spending an appropriate amount of time engaging with adolescents, and gauging their maturity and interests, may help to support the developmental appropriateness of sessions, and prevent adolescents from feeling as though sessions are too childish.

## Discussion

The present study used a mixed method design to investigate how a clown doctor program can impact the mood and experiences of adolescents residing in a psychiatric inpatient unit within a medical centre. Insight was gained into adolescents’ reported levels of fun and interaction with the clown doctors, as well as their feedback on what they liked, disliked and how to improve the program to inform future research and therapy.

Generally, the quantitative findings revealed that the vast majority of adolescents enjoyed their interaction with the clown doctors, with approximately 85% of the sample responding that they have more fun on a day with the clown doctor(s) present than absent. Likewise, all clinical staff reported young people had fun during a clown doctor visit. The positive impacts (i.e., more fun, smiles, laughter and positive interaction) experienced by adolescents was also a consistent observation made by the clown doctors in this study. The benefits of clown doctor visits were reported by clinical staff, with over 96% of staff believing the visit was beneficial. Notably, this finding of clown doctors being able to elicit fun within an adolescent psychiatric setting is especially important as it indicates that the provision of clown doctor programs can be a part of a potential solution to counteract existing problems inherent in psychiatric settings, such as boredom [11, 12] which has been associated with adolescent’s heightened feelings of isolation [12] and depression [41]. Moreover, it has long been acknowledged that children require fun in their treatments and therapeutic environments [42]. For instance, in exposure therapy, it is important for therapists to make the therapy experience fun and enjoyable for children and adolescents, as this helps them build trust and create a positive therapeutic relationship with their therapist, which can facilitate progress towards treatment goals [43]. In brief, our study findings suggest that interventions aimed at promoting fun and enjoyment in psychiatric settings, such as clown doctor programs, are an important component of mental health care for young people.

The quantitative data, as supported by qualitative data, demonstrated that clown doctors were often able to improve the general mood of adolescents and the atmospheric feeling of the ward, from “the usual quiet” to “lots of laughs”. Similarly, over 76% of clinical staff reported a positive change in milieu following a visit. This outcome of elevated mood and atmosphere may carry wider implications for the adolescents’ treatment success at the ward, particularly since the climate or atmosphere of a ward has been demonstrated to have an effect on patient outcomes in inpatient mental health settings [44, 45].

The study’s findings revealed that clown doctors were able to provide a distraction for most adolescents during the clown doctor sessions. Although the success and/or degree of distraction by clown doctors varied amongst adolescents, the type of distraction commonly described was one of an escape from problems. This distraction technique has also shown its success in paediatric research, before medical procedures, whereby reducing preoperative anxiety is the focus [13, 19, 21, 46, 47]. As such, the therapeutic role of distraction in mental health contexts is thought to operate by providing short-term relief from overwhelming or negative feelings and thoughts [48, 49].

The qualitative data elucidated the quantitative data by highlighting the experiences which were particularly fun. Most adolescents were found to have liked and enjoyed the props. In particular, props including the use of bubbles and balloons and activities and games such as table tennis and musical instruments, that were introduced and lead by the clown doctors were described as particularly fun. The perceived success of props, activities and games were also shared by the survey responses of clown doctors; however, a few adolescents did express the need for more creativity, novelty and variety regarding activity and game content.

Adolescents described how clown doctors elicited a sense of respect, privacy and space by giving adolescents an opportunity to complete tasks on their own without forcing participation. As the literature highlights that adolescents generally report feeling powerlessness and/or a lack of autonomy in in-patient settings [6–8], this finding is important in demonstrating that clown doctor sessions allowed the adolescents to maintain some control and autonomy that they may not experience in other aspects of staying in an in-patient ward. Furthermore, whilst adolescents provided mixed responses about whether the humour and jokes told by the clown doctors were age-appropriate and/or funny, most adolescents expressed that they were able to have a mature conservation and develop a positive connection with the clown doctors. Interestingly, the majority of the clown doctors reported similar self-evaluations, that they too recognised and felt that their jokes were not always successful and that their strengths during visits came from conversing and building rapport with the adolescents. Collectively, these preliminary findings suggest that the employment of clown doctors in a psychiatric inpatient setting is beneficial and well-received by many adolescents, and that most clown doctors have a sense of what is and is not effective in positively engaging the adolescents.

Whilst many adolescents expressed that they enjoyed the clown doctor sessions and emphasised there was nothing they would change about the session and/or did not want it to change, it should be noted that there were some adolescents in this study who did not report any positive experiences in their interaction with the clown doctors. Indeed, a small number of adolescents reported negative experiences with the clown doctors, including feelings of exclusion from some clown doctor activities, level of noise being too overwhelming, and/or clown doctors trying too hard to elicit laughter or initiate conversation with adolescents. These concerns were recognised by the clown doctors in their own self-evaluations and were noted as techniques that did not successfully engage the adolescents. A minor area of improvement missed or not mentioned in survey responses by clown doctors, but had been suggested by some adolescents, related to the physical appearance (e.g., makeup and outfits) of the clown doctors being too clown-like, immature and stereotypical of a clown doctor for children as opposed to adolescents. In sum, it can be argued that future clown doctors and intervention programs may benefit from applying the feedback provided from adolescents in this study, particularly given that most of the suggested improvements are seemingly minor in nature and largely modifiable with some personal self-reflection and/or training. Further, although clown doctors can be a valuable addition to a patient’s treatment plan, it is essential for healthcare providers to consider the individual needs and preferences of each patient before including them in such sessions. In our study, we excluded adolescents who were deemed unfit by clinical staff to participate in the clown doctor session due to their current mental state or a condition that might impair their capacity to voluntarily participate. These measures were taken to ensure that patients who may be frightened or negatively impacted by clown visits were not exposed to them. Therefore, to ensure the safe and effective use of clown therapy in psychiatric settings, healthcare providers must exercise caution and tailor interventions to the unique circumstances of each patient.

The present findings suggest that clown doctor programs can be appropriately implemented in adolescent psychiatric inpatient settings with positive effects similar to those found in paediatric [13, 16, 18–24], adult [25–29], and elderly [30–32] research. While tailored clown doctor services for specific age groups currently exist – for instance, the Humour Foundation has a clown doctor program for children and a program for the elderly – similar tailored services for adolescents and or adults do not currently exist in Australia. The qualitative components of the present study, including feedback from adolescents, highlight a number of areas that should be developed to ensure clown doctor programs are effective and age-appropriate for adolescents. These outcomes should be used to help educate, inform and shape the future training of clown doctors for adolescents.

The study collected both qualitative and quantitative data, which is a strength given the early stages of research in this area. Both clown doctor and adolescents’ responses were collected, providing a basis for comparison of perspectives. Notably, both adolescents and clown doctors confirmed each other’s reports, and the quantitative data triangulated the qualitative data, in that most participants noted positive experiences. As a preliminary study, the research has a few limitations. The study was limited to one adolescent psychiatric unit, therefore results may not be generalisable to other units. Bespoke measures designed by the research team with unknown psychometric properties may be a limitation, and further research to test the psychometric properties of the measures is warranted. Future studies may also wish to take pre- and post- measures of variables such as anxiety and mood state using standardized measures. As this study demonstrated that adolescents staying in an in-patient psychiatric ward largely enjoyed the experience of the clown doctor program, randomised controlled trials are an important next step to evaluate the effectiveness of clown doctor programs in supporting therapeutic outcomes of psychiatric wards.

## Summary

The findings of the current study expand the literature on the impacts of clown doctors in an adolescent psychiatric setting. Adolescents reported heightened levels of fun, mood and feeling following the session with the clown doctors. Similarly, clown doctors highlighted the positive impacts on adolescents from their perspective, including heightened fun, improvement of feeling in the room and success of the visit. Preliminary findings highlight the content and outcomes of the session were enjoyed by adolescents, contributing to the positive responses by adolescents. Future clown doctors may benefit from training to further understand the challenges experienced by adolescents in a psychiatric ward and adapting their content to suit an adolescent audience. Learning skills such as knowing when to approach an adolescent and tailoring future sessions to the developmental desires of this group could improve future clown doctor sessions.

## Data Availability

Unfortunately, the data is not available for sharing at this time.
